# Supplementary material for: The Effectiveness and Safety of Wu Tou Decoction on Rheumatoid Arthritis—A Systematic Review and Meta-Analysis
Source: Healthcare (Basel). 2024 Aug 31;12(17):1739. doi: 10.3390/healthcare12171739 (PMC11395211; doi:10.3390/healthcare12171739)
Supplement: Supplementary file 1 [file healthcare-12-01739-s001.zip › healthcare-3132410-supplementary.pdf]

Supplementary S1. The results of sensitivity analysis

Type of WTD	Item	Studies	Data			
			MD or RR	Minimum CI	Maximum CI	Heterogeneity (%)
Mono therapy	ER	The origin results of SR (Figure 2)	1.25	1.14	1.37	48
		Li 2015	1.26	1.13	1.4	74
		#Wei 2010	1.32	1.16	1.49	24
		#Liu 2013	1.17	1.05	1.31	0
	ESR	The origin results of SR (Figure 3a)	24.46	20.72	28.2	N/A
		Li 2015	N/A	N/A	N/A	N/A
	CRP	The origin results of SR (Figure 3b)	3.11	0.66	5.57	29
		#Li 2015	13.87	-4.05	31.79	N/A
		Wang 2016(1)	2.91	0.44	5.38	N/A
Combination therapy	ER	The origin results of SR (Figure 5)	1.25	1.18	1.33	0
		Chen 2015(2)	1.25	1.18	1.33	0
		Hu 2016	1.26	1.18	1.33	0
		Huang 2012	1.25	1.18	1.33	0
		Li 2013	1.25	1.18	1.33	0
		Li 2016	1.25	1.18	1.33	0
		Li 2022	1.25	1.17	1.33	0
		Peng 2019	1.25	1.18	1.34	0
		Wang 2017	1.25	1.18	1.33	0
		Zheng 2016	1.25	1.17	1.33	0
		Huang 2008	1.27	1.19	1.35	0
		Liu 2015	1.26	1.19	1.34	0
		Bai 2020	1.23	1.16	1.31	0
		Zhou 2018	1.26	1.18	1.34	0
	ESR	The origin results of SR (Figure 7a)	9.66	8.88	10.43	88
		Li 2022	10.66	9.8	11.51	83
		Wang 2017	9.7	8.92	10.48	90
		Peng 2019	10.4	9.51	11.29	88
		Bai 2020	9.75	8.96	10.54	90
		Li 2018	9.69	8.91	10.47	90
		Zhou 2018	9.66	8.88	10.45	90
		Zheng 2016	9.48	8.66	10.29	90
		#Hu 2016	7.55	6.58	8.52	65
		Huang 2012	9.59	8.81	10.37	90
	CRP	The origin results of SR (Figure 7b)	6.25	5.75	6.74	92
		Bai 2020	6.26	5.75	6.76	93
		Hu 2016	6.57	6.06	7.09	91
		Li 2018	6.3	5.8	6.79	93
		Li 2022	5.53	4.93	6.13	91
		Peng 2019	5.51	4.95	6.06	89
		Wang 2017	6.24	5.74	6.74	93
		Zheng 2016	6.24	5.73	6.74	93
		#Zhou 2018	7.29	6.71	7.87	87

Abbreviations: CI, confidence interval; CRP, C-reactive protein; ER, Effective rate; ESR, Erythrocyte sedimentation rate; MD, Mean difference; RR, Risk ratio; SR, Systematic review; WTD, Wu tou decoction.

Through the sensitivity analysis, we found studies whose deletion significantly changed the results and marked them with a '#'.
